# Can Samba and Forró Brazilian rhythmic dance be more effective than walking in improving functional mobility and spatiotemporal gait parameters in patients with Parkinson’s disease?

**DOI:** 10.1186/s12883-020-01878-y

**Published:** 2020-08-18

**Authors:** Marcela dos Santos Delabary, Elren Passos Monteiro, Rebeca Gimenes Donida, Mariana Wolffenbuttel, Leonardo Alexandre Peyré-Tartaruga, Aline Nogueira Haas

**Affiliations:** 1 Federal University of Rio Grande do Sul, Street Felizardo, 750, Jardim Botânico, Porto Alegre, Rio Grande do Sul Brazil; 2 Federal University of Pará, Street Guamá, Belém, Pará Brazil

**Keywords:** Parkinsonian disorders, Dance therapy, Rehabilitation, Kinematics, Locomotion, Quality of life

## Abstract

**Background:**

Parkinson’s disease (PD) causes motor and nonmotor disorders in patients. Unlike aerobic training, potential adaptations from the practice of dance are less understood in PD, particularly compared with better known exercise modes. This study aimed to verify and compare the effects of a Brazilian dance program, inspired by Samba and Forrró rhythms, and a walking program on functional mobility and spatiotemporal gait parameters in patients with PD.

**Methods:**

Eighteen participants with PD were divided into a dance group (DG) and a walking group (WG) and were assessed before and after an intervention period of 24 1-h sessions, performed twice per week for 12 weeks. The timed-up-and-go test (TUG) and walking kinematics at self-selected speed (SSS) and fast speed (FS) were determined. The generalized estimating equation method was used to compare the DG and WG pre- and post-intervention and to evaluate the group*time interaction (α <  0.05).

**Results:**

Both groups demonstrated a significant improvement in TUG test at SSS (*p* = 0.02; effect size [ES] = 0.42) and FS (*p* = 0.02; ES = 0.24). In general, spatiotemporal parameters remained unchanged, except at SSS, in which the DG increased the stride frequency (*p* = 0.011; ES = 0.72). At FS, the swing time demonstrated a significant group*time interaction (*p* <  0.001; ES = 1.10), in which the two groups exhibited different behaviors: DG decreased (*p* = 0.015) and WG increased (*p* = 0.012).

**Conclusions:**

Functional mobility improved similarly in both groups. The results suggest that a 12-week program of Brazilian dance was sufficient to produce improvements in functional mobility and gait in individuals with PD.

**Trial registration:**

This study is registered with the International Clinical Trial Registry under number NCT03370315. Registered December 28, 2017 - Retrospectively registered.

## Background

Parkinson’s disease (PD) is the second most prevalent neurodegenerative disease worldwide. In developed countries, approximately 1% of the population aged > 65 and 3–5% of those aged > 85 years are affected by the disease [1]. PD causes changes in the activity of dopaminergic cells in the substantia nigra, leading to motor and nonmotor symptoms [2, 3], including clinical and functional symptoms and locomotion disorders [4, 5].

The motricity of individuals with PD is affected by symptoms such as muscle stiffness, rest tremor, akinesia and bradykinesia, postural instability, gait disturbances, and specific balance changes [5, 6]. Gait is essentially an automatic activity in individuals without neurological disorders. Those with PD, however, lose gait automatism due to changes and impairment of dopamine and other striatal neurotransmitters [2, 5, 7]. In relation to healthy subjects, PD people have greater variability and difficulty regulating the spatiotemporal gait parameters, reducing step length (SL) and step frequency (SF), while increasing double foot support time [8–10], thus increasing the risk of falls [11]. Gait changes are the major limitations caused by the disease in this population, impairing autonomy, activities of daily living and, consequently, the quality of life [11–13].

Research data indicate that, together with drug therapy, regular physical activity can promote improvement in the clinical and functional symptoms of individuals with PD [7, 13, 14]. In this context, walk training is a highly typical exercise because it is considered to be a functional activity and, therefore, highly recommended for those with PD [10, 15, 16]. This type of training assists in functional mobility and improvement in spatiotemporal gait parameters [13, 16].

Dance is another type of practice that has emerged as a complementary therapeutic strategy to promote physical, psychological, and social benefits [17]. Similar to walking, dance practices require constant weight transfer between the legs at different temps according to musical stimuli [18] and changes in direction. In addition to being accessible to those with PD, dance classes can help improve motor parameters [19], increase quality of life, stimulate socialization, and provide greater motivation to adhere to body movement practices [20, 21].

Among studies that have suggested dance as a non-pharmacological therapeutic practice for individuals with PD, the most frequently studied style is the “tango” [3, 22, 23]. Similar to other dance modalities, the tango requires movement initiation and completion, plus rapid changes in body direction and displacement [21] dynamic balance, and constant adjustments to the environment and space, as well as using different speeds and rhythms [18, 23].

However, little is known about the effects of Brazilian dance styles on the motor and non-motor aspects of PD. Samba and Forró are two Brazilian dances, danced in pairs, originating in the customs of African songs and dances present in Brazilian popular culture [20, 24–26]. Both musical rhythms involve percussive instruments, which generate a rhythmic pulse through the binary (Samba) and quaternary (Forró) measures. In dance, the presence of music can act as an auditory track capable of determining initiation [22], rhythm, and step length [13], as well as referring to subjective aspects of expressiveness according to the participants’ musical tastes [22].

Despite the benefits of dance practice indicated in research data [17, 19–23, 27–30], results regarding improvements in functional mobility and motor symptoms have varied widely. To our knowledge, there are no studies that compare the effects of dance and walking in patients with PD. Moreover, there are no studies investigating the effects of dance on Parkinson’s locomotion using three-dimensional analysis.

Thus, the aim of this study was to verify and compare the effects of a Brazilian dance program, inspired by Samba and Forrró rhythms, and a walking program on functional mobility and spatiotemporal gait parameters in patients with PD. We hypothesized that Samba and Forró Brazilian rhythmic dance can be more effective than walking in improving functional mobility and spatiotemporal gait parameters in patients with Parkinson’s disease.

## Methods

### Design and setting

The present investigation was a non-randomized clinical trial that applied a dance intervention protocol with 24 classes inspired by Samba and Forró Brazilian rhythms and compared it with an intervention based on walk training. This study was approved by the Research Ethics Committee of the Federal University of Rio Grande do Sul (Brazil, CAAE 68383317.4.0000.5347) and adhered to the principles of the Declaration of Helsinki. It was registered with the *International Clinical Trial Registry* (NCT03370315).

### Participants

Sample size was calculated based on a non-randomized experimental study that applied the timed up and go (TUG) test to assess functional mobility after intervention with a Tango dance program for PD patients [22]. Accordingly, a sample of 18 participants was reported to be necessary to meet the objectives of the present study. Considering the possibility of drop outs, the sample size was increased by 10%, totaling 20 participants, with 10 participants allocated to each group.

The GPower 3.1 software was used to calculate the sample size, with an alpha level of 0.05 and a power of 90%, a standard deviation of the primary outcome of 0.4 s, and a minimum detectable difference of 1 s, based on standard deviations and differences between the means obtained in the study by Hackney et al. [22].

The inclusion criteria for participants in this research were: being an adult of either sex; ≥ 50 years of age; with a clinical diagnosis of PD provided by a neurologist for ≥1 year according to the criteria from the Queen Square Brain Bank [31]; staging between 1 and 3 of the Hoehn and Yahr Scale (H&Y); undergoing medical treatment for PD with regular use of anti-parkinsonian drugs; able to walk independently; and accepting to participate in the research by signing the informed consent form. The exclusion criteria were: having risk factors, such as recent surgery, deep brain stimulation, and other associated neurological or chronic diseases; missing more than 25% of the classes; or changing the established exercise routine.

Participants were recruited by the researchers via social media, flyers in Parkinson’s Association and Health services, and telephone calls using the waiting list for other activities for Parkinson’s in the School of Physical Education, Physiotherapy and Dance of *Universidade Federal do Rio Grande do Sul* (ESEFID/UFRGS), Brazil.

The subjects included 18 participants with PD, who were divided non-randomly into two groups: dance group (DG [*n* = 12]), which participated in a dance program based on Samba and Forró Brazilian rhythm; and a walking group (WG [*n* = 6]), which participated in a walking program. Participants could choose their preferred activity program at the beginning of the study. All the participants received and signed an informed consent form agreeing to participate in this research.

### Intervention protocols

This study involved two different interventions, each lasting 12 weeks. The interventions were held at the ESEFID/UFRGS. Both groups received an intervention protocol explaining the specificities of each activity to be performed during the 24 sessions, each lasting 1 h, twice per week on alternating days. After the end of the intervention, the participants were invited to continue attending one of the two activity groups, dancing or walking.

#### Dance program

The dance program was conducted in an appropriate room for dance classes with mirrors, chairs, and a barre. Dance classes were taught by a qualified dance teacher, with a University degree in Dance. Undergraduate dance students acted as monitors during the classes.

Table 1 summarizes a version of this protocol in four parts. The intensity of the classes was measured based on the music tempo [32] and beats per minute.

**Table 1:** Dance classes

Class Part	Time	Content	Tempo	BPMs
**Part 1**	15 min	Joint warm-up, stretching and body sense, sitting on chairs in a circle.	Moderato	80–120
**Part 2**	15 min	Strength, balance, and rhythm exercises, walking and clap hands with the support of the barre.	Moderato to Allegro	96–168
**Part 3**	15 min	Exercises in front of the mirror with movements thought the room inspired by samba e do forró basic steps (Brazilian ballroom dance). Exploration of movements in the rhythm of the music. Exercises in pairs (couples).	Moderato to Allegro	104–192
**Part 4**	15 min	Rhythmic and playful exercises with walking and clap hands in a circle. Ludic activities that stimulate socialization or visual cues with walking, motor coordination, rhythm, improvisation, and creativity. Final cool down in a circle.	Moderato to Allegro	104–192

#### Walking program

The walking program was performed outdoors on a 400 m track to facilitate the calculation of the distance covered in each training session. Walking classes were taught by qualified teachers, with a University degree in Physical Education. Undergraduate physical education students acted as monitors during the classes.

Table 2 summarizes the three parts of the class. The second part of the class was prepared individually for each student. Individual and daily training of the participants was part of the general training cycle, which was elaborated upon based on the specific functional capacity of each participant and measured using the 6-min walk test before the classes began. Each training program had an individualized speed variation for each participant (self-selected speed [SSS, intermediate] and fast speed [FS]), and the distance was determined by the number of laps traveled.

**Table 2:** Walking class

Class Part	Time	Description
**Part 1**	15 min	Joint warm-up and muscular stretching in a circle.
**Part 2**	30 min	The principle part of the class, individual daily walk training according to the specific functional capacity of each participant. Volume and intensity vary according to the distance to be traveled and speed.
**Part 3**	15 min	Final cool-down (stretching and massage) in a circle.

### Outcome measures

Before beginning the interventions, to characterize the sample, the participants completed an anamnesis with personal information, and the Unified Parkinson’s Disease Rating Scale (UPDRS) III and the Hoehn & Yahr (H&Y) scale were applied.

Table 3 summarizes the measured outcomes and other instruments used for data analysis. All the instruments used are considered the gold standard for the study population.

**Table 3:** Outcomes and instruments

Outcomes	Instruments
FUNCTIONAL MOBILITY SSS and FS	Timed Up and Go(TUG) test
GAIT ANALYSIS Spatio-temporal gait parameters: SSS, FS, SF. StT, SwT, SL AND DSP	Gait Kinematic analysis using with a synchronized system composed of six infrared cameras (BTS SMART DX 7000 System) with resolution of 1 Megapixel, accuracy < 0.3 mm (calibration volume of 4 × 3 × 3 m), acquisition rate of 100 Hz, synchronized with one Digital videocamera (BTS eVIXTA) 1.9 Megapixel with acquisition rate up to 100 Hz [33]

Note: *SSS* Self-selected speed, *FS* Fast speed, *SF* step frequency, *StT* stance time, *SwT* swing time, *SL* step length and *DSP* double support phase

**Fig. 1:**
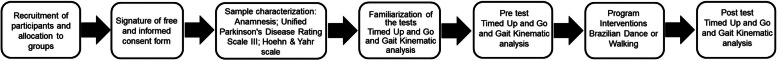
Study timeline

For both groups, laboratory test procedures were used to improve and monitor adherence to the intervention protocols. Explanation and familiarization of the tests were administered before data collection and, during the tests, the evaluator used verbal commands. Throughout the intervention period, the participants were instructed to maintain their usual prescribed medications, eating and exercise habits. The tests were held at the ESEFID/UFRGS. In addition, all procedures were performed in the “ON” state, between one and three hours after taking their anti-parkinsonian medications. When performing the tasks, the participants showed no signs of dyskinesia. To control possible motor fluctuations, we took pre and post intervention measurements at the same time of the day. The researchers responsible for the classes were different from the researchers who applied the tests and analyzed the results. Figure 1 illustrates the study timeline.

### Statistical analyses

Data from the sample are presented descriptively, and group means were compared to reveal differences at baseline. Data normality was verified using the Shapiro-Wilk test. The Wilcoxon test for non-parametric data was used for data that were non-normally distributed, while a parametric technique (paired T-test) was used for variables that were normally distributed.

Outcome results were analyzed using the generalized estimating equation (GEE) method to compare between the pre- and post-intervention DG and WG. The GEE statistical model was used to test different covariance matrices to provide regression coefficients and standard errors [34, 35]. The models were calculated separately for each variable. Each variable that could potentially influence the behavior of the results was tested separately as a covariate in each model. Time effects, group, and group*time interaction were analyzed. A Bonferroni post-hoc test was used to identify the differences between the means for all variables. In addition, the effect size (ES) between pre- and post-intervention from Cohen’s d are presented as mean and corresponding 95% confidence interval, according to the qualitative classification, as follows: small, between 0.2 and 0.5; medium, between 0.5 and 0.8; and large, ≥ 0.8. SPSS version 20.0 (IBM Corporation, Armonk, NY, USA) was used to analyze the data. The significance level adopted for both tests was α < 0.05.

## Results

**Fig. 2:**
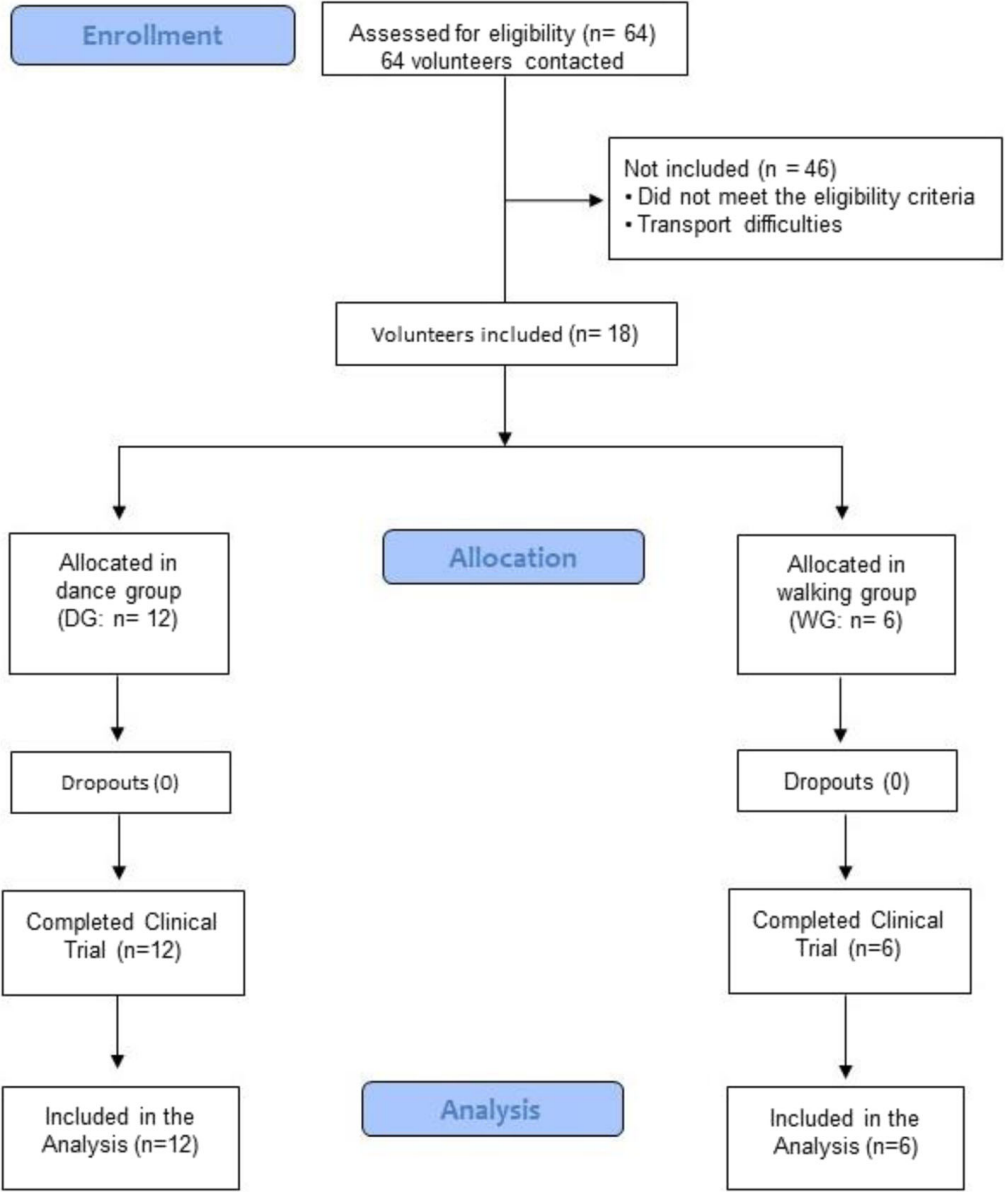
Flowchart CONSORT Sample Selection Process and Participants Inclusion

The sample consisted of 18 participants (DG [*n* = 12]; WG [*n* = 6]). The sample selection process, from recruitment to final study analysis, is illustrated in the flow diagram presented in Fig. 2.

The reasons reported for non-entry of participants in the research included difficulty in travelling to the intervention site and unavailability during the class schedule. There were no dropouts throughout the intervention. After completing the study, the majority of students requested to continue their activities in university extension projects, demonstrating adherence to body practices.

The groups demonstrated a statistically significant difference at baseline only in the motor symptoms evaluation, as measured by the application UPDRS III, in which the DG exhibited less motor impairment than the WG (*p* = 0.016) (Table 4).

**Table 4:** Descriptive statistics, with central tendency values: mean for parametric data^a^ and median for nonparametric data^b^; dispersion measures: standard deviation (SD) for parametric data and standard error (SE) for nonparametric datab; Confidence Interval (CI); difference between (p) dance (DG) and walk (WG) groups at the initial moment of the research; and sample characterization data

Outcomes	DG (***n =*** 12)	WG (***n*** = 6)	***P***
Age	68.6 ± 6.7 [64.3–72.8] ^**a**^	64.2 ± 4.9 [59.0–69.3] ^**a**^	0.174
Body Mass (kg)	75.2 ± 11.3 [68–82.3] ^**a**^	75.2 ± 13.2 [61.3–89] ^**a**^	1.000
Height (m)	1.60 ± 0.08 [1.55–1.65] ^**a**^	1.64 ± 0.05 [1.59–1.69] ^**a**^	0.266
Diagnosis Time Disease	3.5 ± 1.3 [2–7.8] ^**b**^	5.5 ± 2.2 [1.9–13.1] ^**b**^	0.151
H&Y	1.5 ± 0.2 [1.3–2.4] ^**b**^	2.5 ± 0.3 [1.5–3.1] ^**b**^	0.180
TUG SSS (s)	11.7 ± 2.5 [10.1–13.3] ^**a**^	11.2 ± 1.7 [9.4–13] ^**a**^	0.677
TUG FS (s)	9.3 ± 0.6 [8.2–10.7] ^**b**^	9.3 ± 0.4 [8.2–10.4] ^**b**^	0.820
UPDRS III	13.5 ± 7.4 [8.8–18.2] ^**a**^	23.8 ± 8.2 [15.2–32.5] ^**a**^	0.016*
SSS (m/s)	1.02 ± 0.2 [0.9–1.1] ^**a**^	1.08 ± 0.2 [0.9–1.3] ^**a**^	0.464
Sex	9 women / 3 men	2 women / 4 men	NA
Clinical Symptoms			
ȀInstability / lack of balance	8 (66.6%)	4 (66.6%)	NA
Tremor	10 (83.3%)	5 (83.3%)	NA
Postural change	2 (16.6%)	1 (16.6%)	NA
Rigidity	9 (75%)	5 (83.3%)	NA
Bradykinesia	12 (100%)	6 (100%)	NA
Dyskinesia	2 (16.6%)	1 (16.6%)	NA
Falls History	6 (50%)	3 (50%)	NA
**DP specific medication**			
Levodopa – Cardidopa	2 (16.6%)	0 (0%)	NA
daily dose (mg)	1650 (1200–2100)	0 (0–0)	NA
Prolopa	10 (83.3%)	6 (100%)	NA
daily dose (mg)	585 (250–1000)	545 (188–1000)	NA
**Physical Exercise Practice**	8 (66.6%)	4 (66.6%)	NA

Note: *UPDRS* Motor part of the Unified Parkinson’s Disease Scale, *H&Y* Hoehn & Yahr, scale, *TUG* Timed up and Go test, *SSS* Self-selected speed, *mg* Milligram

^a^ indicates that the data has a parametric distribution. Presented as mean ± SD [CI]; ^b^ indicates that the data have a nonparametric distribution. Presented as median ± SE [CI]; * indicates statistically significant difference between groups. NA indicates not applicable

### Outcomes

Functional mobility outcomes and spatiotemporal gait parameters were compared between the DG and WG, time (pre- and post-intervention), and group*time interaction.

For some outcomes, disease duration and staging according to the H&Y scale were adopted. The TUG test functional mobility variables at SSS and FS and the spatiotemporal parameters SF, SL and step time (StT) at SSS were analyzed using the disease time covariate. Swing time (SwT) at SSS was compared without any covariates. The spatiotemporal parameters SF, SL, StT, and SwT at FS were used in addition to disease duration, with H&Y as a covariate in the analysis.

In cases in which there was a significant difference in the group*time interaction, the Bonferroni post hoc was applied, for which the means of each group were observed at each time point to identify where the difference was specifically.

#### Functional mobility: TUG test

Both groups demonstrated significant improvements in functional mobility after the intervention period, compared with pre-intervention, reducing the task execution time, both at SSS (*p* = 0.002; ES = 0.42) and FS (*p* = 0.002 ES = 0.24) (Table 5).

**Table 5:** Mean values and standard error (SE) of the UPDRS III and TUG variables in the DG and WG groups at the PRE and POST intervention moments; and the difference (p) regarding group, time and group*time interaction, Effect Size and Confidence Interval (IC)

Variables	Intervention	PRÉ		PÓST		***p*** – valor			Effect Size [CI]
		Mean ± SE		Mean ± SE		Group	Time	Group*Time	
TUG SSS (s)	DG	11.70	0.7	10.20	0.5	0.892	0.002*	0.062	0.42 [-0.60 to 1.37]
	WG	11.22	0.6	10.84	0.4				
TUG FS (s)	DG	9.46	0.6	8.58	0.5	0.864	0.002*	0.175	0.24 [-0.76 to 1.21]
	WG	9.29	0.4	8.94	0.3				

*TUG* Timed up and Go Test, *SSS* self-selected speed, *FS* fast speed, *UPDRS III* Motor part of the Unified Parkinson's Disease Scale. *indicates statistically significant difference (*p* < 0.05)

#### Gait analysis: spatiotemporal gait parameters of gait at SSS

Regarding kinematic analysis of gait at SSS, a significant difference was found between the groups in SwT (*p* = 0.011; ES = 1.50) (Table 6).

**Table 6:** Mean values and standard error (SE) variables of spatiotemporal gait parameters in the SSS in the DG and WG groups at the PRE and POST intervention moments; and the difference (p) regarding group, time and group*time interaction, Effect Size and Confidence Interval (IC)

Variables	Intervention	PRÉ		PÓST		***p*** – valor			Effect Size [CI]
		Mean ± SE		Mean ± SE		Group	Time	Group*Time	
Velocity (m/s)	DG	1.02	0.05	1.10	0.07	0.762	0.960	0.960	0.09 [−0.89 to 1.07]
	WG	1.08	0.06	1.08	0.06				
SF (steps/min)	DG	111.48^#^	3.05	118.50^#^	3.63	0.196	0.009*	0.011**	0.72 [−0.34 to 1.67]
	WG	110.00	2.46	110.10	2.05				
SL (m)	DG	0.54	0.02	0.57	0.03	0.380	0.052	0.129	0.25 [−0.90 to 1.37]
	WG	0.59	0.02	0.59	0.02				
StT (s)	DG	0.73	0.02	0.69	0.03	0.869	0.215	0.074	0.35 [−0.82 to 1.46]
	WG	0.71	0.02	0.72	0.03				
SwT (s)	DG	0.36	0.009	0.34	0.007	0.011*	0.054	0.144	1.50 [0.30 to 2.46]
	WG	0.38	0.008	0.37	0.01				
DSP (%)	DG	17.10	0.80	16.69	1.00	0.061	0.502	0.891	0.68 [−0.37 to 1.63]
	WG	14.83	0.80	14.60	0.80				

Note: *SF* step frequency, *SL* step length, *StT* stance time, *SwT* swing time and *DSP* double support phase

*indicates statistically significant difference (*p* < 0.05); **indicates group*time interaction, ^#^indicates where is the difference found in group*time interaction after Bonferroni post hoc application

It is noteworthy that the SF presented statistically significant difference in the interaction group*time (*p* = 0.011; ES = 0.72), and, from Bonferroni post-hoc, a significant difference was observed between the DG and WG groups at post-intervention (*p* = 0.044) and between pre- and post-intervention of the DG (*p* = 0.006). Due to this interaction, SF in the DG increased significantly after the 24 dance sessions (pre-intervention = 111.48 ± 3 steps/min; post-intervention = 118.5 ± 4 steps/min) (Table 6).

#### Gait analysis: spatiotemporal gait parameters at FS

Regarding kinematic analysis of gait at FS, there was a significant decrease in StT (*p* = 0.009; ES = 0.16) and double support phase (DSP) (*p* < 0.001; ES = 0.39) in both groups after the intervention period (Table 7).

**Table 7:** Mean values and standard error (SE) variables of spatiotemporal gait parameters in the FS in the DG and WG groups at the PRE and POST intervention moments; and the difference (p) regarding group, time and group*time interaction, Effect Size and Confidence Interval (IC)

Variables	Intervention	PRÉ		PÓST		***p*** – valor			Effect Size [CI]
		Mean ± SE		Mean ± SE		Group	Time	Group*Time	
Velocity (m/s)	DG	1.43	0.10	1.48	0.07	0.477	0.464	0.464	0.23 [−0.77 to 1.20]
	WG	1.53	0.08	1.53	0.06				
SF (steps/min)	DG	136.05	5.70	142.23	4.30	0.544	0.563	0.114	0.62 [−0.43 to 1.57]
	WG	137.10	4.70	134.30	2.50				
SL (m)	DG	0.64	0.04	0.63	0.03	0.379	0.295	0.110	0.76 [−0.30 to 1.71]
	WG	0.66	0.03	0.70	0.03				
StT (s)	DG	0.60	0.03	0.55	0.02	0.940	0.009*	0.250	0.16 [−0.84 to 1.12]
	WG	0.58	0.03	0.56	0.02				
SwT (s)	DG	0.32^#^	0.01	0.30^#^	0.01	0.544	0.814	< 0.001**	1.10 [−0.02 to 2.05]
	WG	0.31^#^	0.01	0.33^#^	0.01				
DSP (%)	DG	14.78	1.00	13.75	0.80	0.790	< 0.001*	0.113	0.39 [−0.63 to 1.34]
	WG	15.22	1.20	12.72	1.00				

Note: *SF* step frequency, *SL* step length, *StT* stance time, *SwT* swing time and *DSP* double support phase

*indicates statistically significant difference (*p* < 0,05); **indicates group*time interaction, ^#^indicates where is the difference found in group*time interaction after Bonferroni post hoc application

It is noteworthy that SwT revealed a statistically significant difference in relation to the group*time interaction (*p* < 0.001; ES = 1.10). From Bonferroni post-hoc, it was observed that the groups exhibited different behaviors: DG showed decreased SwT (pre-intervention = 0.32 ± 0.01 s; post-intervention = 0.30 ± 0,01 s; *p* = 0.015) after 24 dance sessions, while the WG showed increased SwT (pre-intervention = 0.31 ± 0.01 s; post-intervention = 0.33 ± 0.01; *p* = 0.012) (Table 7).

## Discussion

The aim of the present study was to verify and compare the effects of a Brazilian dance program, inspired by Samba and Forrró rhythms, and a walking program on functional mobility and spatiotemporal gait parameters in patients with PD. Both interventions resulted in significant improvements regarding functional mobility at both speeds (i.e., SSS and FS) after the 12-week intervention, rejecting the first hypothesis of the study. In the spatiotemporal parameters of gait at SSS, a difference was observed only in SF, indicating an improvement in the DG and a difference between groups DG and WG in SwT. FS, StT, and Double Phase Support (DPS) decreased in both groups, and SwT reduced in DG and increased in WG. Thus, the study hypothesis was partially accepted.

Significant gains in functional mobility are relevant to the study population because improvements in this regard may promote benefits in the independence and autonomy of individuals with PD [22], as well as assist in the prevention of falls [5, 14]. Delabary et al. pointed out that the practice of dance presents better responses in the functional mobility of individuals with PD compared with other types of exercise, such as physical therapy and/or self-directed exercises [19]. However, in the present study, dance was as effective as walking in improving functional mobility.

Regarding gait mechanics, spatiotemporal parameters are related, and the behavior of one can interfere with another during the stride cycle. From the perspective of kinematic analysis of gait at SSS, there was a significant increase in the SF in the DG after the intervention. This increase in the number of steps per minute may represent an increase in internal work (winter) of muscles during the gait analysis [36, 37].

On analyzing SL, no significant difference was found, suggesting minimal changes after both interventions, which may also represent maintenance of the external work (Wext) of locomotion in individuals with PD [36, 37]. Thus, the significant increase in FS in the DG does not characterize festination, suggesting that the DG appeared to better adjust to the mechanical parameters of locomotion [38]. Our findings demonstrate the benefit of dance practice for improving the biomechanical quality of gait of these individuals, which may reduce the risk for falls, agreeing with previous studies [30, 38].

SwT at SSS demonstrated divergent changes between the groups, indicating a different behavior between them according to this variable. The reduction in SwT is directly related to the increase of SF and, consequently, velocity [39]. Thus, at FS, the DG demonstrated a decrease in SwT, while the WG demonstrated an increase in this same variable. A smaller SwT may be related to a faster walk, whereas a larger SwT may represent a slower walk or less double-support walk [39, 40]. Although the DG had the lowest SwT values, both groups exhibited a downward trend, as confirmed by the large ES (1.50).

At FS, there was a significant decrease in StT and DSP in both groups, demonstrating a benefit of both protocols in gait improvement in patients with PD. Individuals with PD tend to exhibit a higher StT during the stride compared with healthy individuals due to the longer time of double ground support [7, 41]. A smaller StT represents better motor control, symmetry, and joint stability during walking, directly inferring walking economy [39]. These findings infer that the dance and walk program protocols contributed to better symmetry of spatiotemporal gait parameters, which resulted in an optimization of the pendular walking mechanism [36, 39, 40]. Therefore, models of both proposed interventions may contribute to lower energy expenditure [39].

Regarding the relationship between spatiotemporal parameters at FS, the decrease in SwT in the DG was combined with a significant decrease in StT and DSP. In the WG, the increase in SwT was combined with the significant decrease in StT and DSP. This aspect may be related to the tasks performed while dancing, where the demand for the execution of the steps with the rhythm of music demands a smaller SwT compared with the movement of walking. In the Brazilian dance class protocol, the individuals were encouraged to perform body movements in space in various directions (i.e., forward, sideways, backward), weight transfers, and turns [20, 26]. Another aspect to be considered is that the presence of percussive musical instruments, used in the Samba and Forró rhythm, can be considered to be auditory cues to initiate rhythmicity to movement [13, 18, 20, 22].

These findings may be explained by the rhythmicity of the dance, which can stimulate, in a chronic fashion, improvements in the spatiotemporal gait parameters [18]. Studies indicate that rhythmic stimulation can modify the pattern of muscle activation in individuals with PD by adjusting motor control [38, 42, 43].

Findings of the present study are relevant because they highlight the importance of two activities, dance and walking, with different characteristics as complementary therapies for PD. Engagement in the practice of exercise is extremely important for better functional gains in this patient population [13, 15]. In this sense, it is essential to find different activities that can offer benefits to individuals with PD so that they can engage in the one that best ensures well-being and pleasure [14, 30].

Exercise programs contribute to the functional improvement of mobility-related aspects of individuals with PD, especially at SSS and for spatiotemporal gait parameters [10]. However, no studies investigating the effects of dance in this context were found. Thus, results regarding the spatiotemporal aspects of gait found in the present study are relevant to the PD population and the scientific community.

This study has a few limitations. The first is the heterogeneity of the intervention groups (DG and WG), in terms of the number of participants and their baseline motor symptom measurements. In fact, this heterogeneity is a result of the non-randomized nature of the study, which meant we were unable to allocate the ideal number of participants to each group, according to sample size power calculation. However, according to Sterne et al. (2019) similar issues related to post-intervention features occur in randomized trials [44].

The study also has a few strengths. First, the originality of the topic addressed and the promising results obtained with the two interventions, Brazilian Dance and walking. Second, the innovatory use of biomechanical analysis to present the results of the effects of dance in people with PD. And third, the application of these findings in a clinical setting which is of considerable benefit to this population.

Further studies using a randomized clinical trial design and a larger number of participants are, therefore, suggested to better characterize the effectiveness of these interventions.

## Conclusions

Results of the present study demonstrated that the two activities, dance and walking, yielded promising benefits for functional mobility in individuals with PD. In general, spatiotemporal parameters remained unchanged, except at SSS, in which the DG exhibited an increase in SF. Moreover, at FS, SwT demonstrated a significant group*time interaction, in which the groups exhibited different behaviors: SwT decreased in the DG and increased in the WG. The results indicate that a 12-week Brazilian dance program was sufficient to produce improvements in functional mobility and gait in individuals with PD.

The results of this study are a positive resource because they describe the value and/or benefits of two activities with different resources as complementary therapy, allowing the possibility of corporate practices and facilitating or engaging individuals with PD in activities that interest them. It also includes the benefits of the Brazilian dance rhythms Samba and Forró.

## Supplementary information

**Additional file 1.**

## Data Availability

All data generated or analysed during this study are included in this published article and its supplementary information files [see Additional file 1].

## Abbreviations

PD: Parkinson’s Disease
H&Y: Hoehn and Yahr Scale
ESEFID/UFRGS: School of Physical Education, Physiotherapy and Dance of *Universidade Federal do Rio Grande do Sul*
DG: Dance Group
WG: Walking Group
BPMs: Beats per minute
UPDRS III: Unified Parkinson’s Disease Rating Scale III
TUG: Timed Up And Go test
SSS: Self-select speed
FS: Fast speed
SF: Step frequency
StT: Stance time
SwT: Swing time
SL: Step length
DSP: Double support phase
GEE: Generalized Estimating Equations
SPSS: Statistical Package for Social Sciences
SD: Standard deviation
SE: Standard error
CI: Confidence Interval
